# Surveying Potential Vectors of Apple Proliferation Phytoplasma: Faunistic Analysis and Infection Status of Selected Auchenorrhyncha Species

**DOI:** 10.3390/insects12010012

**Published:** 2020-12-26

**Authors:** Stefanie Fischnaller, Martin Parth, Manuel Messner, Robert Stocker, Christine Kerschbamer, Katrin Janik

**Affiliations:** Laimburg Research Centre, Laimburg 6, Pfatten (Vadena), IT-39040 Auer (Ora), Italy; martin.parth@laimburg.it (M.P.); amperspitze@gmail.com (M.M.); robert.stocker@laimburg.it (R.S.); christine.kerschbamer@laimburg.it (C.K.); katrin.janik@laimburg.it (K.J.)

**Keywords:** apple proliferation, ’ *Candidatus* Phytoplasma mali’, Auchenorrhyncha, insect vector, infection rate

## Abstract

**Simple Summary:**

Apple proliferation disease is one of most important threats for European apple cultivation. It is caused by the bacterium ’ *Candidatus* Phytoplasma mali’ which is transmitted by the psyllids *Cacopsylla picta* and *Cacopsylla melanoneura* in South Tyrol. Since no curative treatment is available against the disease, uprooting of infected trees and phytosanitary measures against the known insect vectors are the only strategies to prevent a spread of the pathogen. Interestingly, densities of the known vector insects and disease manifestation in do not always correlate, indicating that other insect vectors might be involved in the transmissive cycle. To elucidate if other insects are involved in ’ *Ca.* Phytoplasma mali’ transmission, a faunistic characterization of the Auchenorrhyncha community of the agroecosystem “apple orchard” was performed and potential vectoring insect species were PCR-analyzed to unravel if they carry the pathogen. Several Auchenorrhyncha species were identified that have never been described before in the territory of South Tyrol, but ’ *Ca.* Phytoplasma mali’ was detected only sporadically in species other than the known vectors. The results provide valuable insights into the Auchenorrhyncha species composition in apple orchards, confirm the prevalent role of the known vectors in the region and are thus an important basis for phytosanitary decision-making.

**Abstract:**

Apple proliferation (AP) is one of the economically most important diseases in European apple cultivation. The disease is caused by the cell-wall-less bacterium ’ *Candidatus* Phytoplasma mali’, which is transmitted by *Cacopsylla picta* (Foerster) and *Cacopsylla melanoneura* (Foerster) (Hemiptera: Psylloidea). In South Tyrol (Italy), severe outbreaks were documented since the 1990s. Infestation rates of AP do not always correlate with the population densities of the confirmed vectors, implying the presence of other, so far unknown, hemipterian vectors. By elucidating the species community of Auchenorrhyncha (Insecta: Hemiptera) at a regional scale, more than 31,000 specimens were captured in South Tyrolean apple orchards. The occurrence of 95 species was confirmed, whereas fourteen species are new records for this territory. Based on the faunistical data, more than 3600 individuals out of 25 species were analyzed using quantitative PCR to assess the presence of AP phytoplasma. The pathogen was sporadically detected in some individuals of different species, for example in *Stictocephala bisonia* Kopp and Yonk (Hemiptera: Membracidae). However, the concentration of phytoplasma was much lower than in infected *C. picta* and *C. melanoneura* captured in the same region, confirming the role of the latter mentioned psyllids as the main insect vectors of AP- phytoplasma in South Tyrol.

## 1. Introduction

Phytoplasmas are plant pathogenic bacteria of the class Mollicutes [1,2] characterized by an obligate, phloem-limited lifecycle. They are transmitted primarily by sap-sucking insects in a persistent and propagative manner [3]. Consequently, the epidemiology of phytoplasma diseases is complex, as it is affected by the interaction between the bacterial pathogen, the insect vector and its plant host [4]. The dissemination of vector-transmitted pathogens is highly correlated to survival and dispersal of the associated vector. Additionally, vector feeding behavior and host-range determine the transmission potential of the respective insect [5,6]. Furthermore, the epidemiological cycle can be complicated by the involvement of more than one insect vector, showing different biology, habitat preferences, and/or distinct transmission capabilities [4,7,8,9]. Additionally, several studies had shown co-adaptions of phytoplasma subtypes and insect vectors, as well as possible events leading to shifts from one insect vector or host plant to another [10,11,12].

’ *Candidatus* Phytoplasma mali’, the causal agent of the quarantine disease Apple proliferation (AP), is among the economical most important pathogens in European apple growing regions [13]. Infected apple trees show profound disturbance in their carbohydrate and hormone metabolism, leading to impaired fruit quality and quantity [14,15,16,17,18]. Further economic losses are caused because growers are obliged by law to eradicate infected trees. In South Tyrol (Northern Italy), one of the largest connected apple growing regions in Europe, severe outbreaks of the disease have occurred since the late 1990s [19]. From 2006 to 2010, more than one million apple trees had to be uprooted in the area of Burgraviato and Val Venosta, the hotspots of AP incidence within South Tyrol [20]. The management of AP is primarily based on insecticide applications during vector presence [21,22]. Since specific vector-targeted insecticide treatments are the only measures at the moment to prevent further spreads or epidemic AP outbreaks, it is crucial to gain detailed knowledge about the vectoring capacity of insect species present in the orchard.

The two psyllid species, *Cacopsylla picta* (Foerster) and *C. melanoneura* (Foerster) (Hemiptera: Psylloidea), have been identified as the main vectors of ’ *Ca*. P. mali’ [23,24,25]. However, the role of *C. melanoneura* in ’ *Ca.* P. mali’ transmission is controversial, and it seems that there are regional differences regarding their transmission efficiency: In Germany, *C. melanoneura* has no relevance as a ’ *Ca*. P. mali’ vector [26], whereas in northeastern and northwestern Italy it plays a predominant role [27,28,29]. In recent years, an intensive monitoring program in South Tyrol revealed low densities of *C. picta*, even if high frequencies of infected trees have been observed [30]. Therefore, it has been speculated that other insects could be involved in ’ *Ca*. P. mali’ transmission.

Insect vectors of phytoplasmas are found exclusively in the order of Hemiptera, suborder Auchenorrhyncha [31,32,33]. Previous surveys showed a natural infection with ’ *Ca*. P. mali’ in field-caught individuals of *Philaenus spumarius* (Linnaeus) (Hemiptera: Aphrophoridae) [34], *Artianus interstitialis* (Germar) (Hemiptera: Cicadellidae) [34], *Stictocephela bisonia* Kopp and Yonk (Hemiptera: Membracidae) [35], *Metcalfa pruinosa* Say (Hemiptera: Flatidae) [36], and *Fieberiella florii* (Stål) (Hemiptera: Cicadellidae) [37]. The transmission capability of the latter has been shown by Tedeschi and Alma [38], but never been confirmed for the other four species.

Hitherto, a limited number of surveys studied the Auchenorrhyncha fauna in South Tyrol [39,40,41,42], and no data on the current situation of species composition in apple orchards and nearby surroundings were available. To provide data regarding the presence of Cicadomorpha and Fulgoromorpha species in apple orchards, a three-year field monitoring survey in the apple growing regions of South Tyrol had been performed, providing an important insight into the biodiversity of this agroecosystem. The detailed faunistic analysis was the basis to identify further, so far unknown vectors that are possibly involved in AP phytoplasma spread. The presence of ’ *Ca*. P. mali’ was analyzed in a subset of species by quantitative PCR (qPCR), focusing on species present in high frequencies in various orchards. In this study, a semi-automated high-throughput quantitative qPCR method was established to analyze AP phytoplasma presence and quantity in thousands of individuals from different insect species.

The aims of this study were to (i) determine Auchenorrhyncha species present in South Tyrolean apple orchards, (ii) use the information to determine a subset of species potentially involved in ’ *Ca*. P. mali’ transmission, (iii) establish a high-throughput PCR method to analyze and quantify ’ *Ca*. P. mali’ in different insect species, and (iv) analyze a high, representative number of individuals of potentially ’ *Ca*. P. mali’ transmitting insects.

## 2. Materials and Methods

### 2.1. Sample Sites

Field surveys were conducted from 2014 to 2016 in apple orchards located in Burgraviato and Val Venosta (South Tyrol, Northern Italy) (see Table S1). Sample sites with and incidence of 0% to 20.1% AP-symptomatic trees per site in 2012/2013 (data provided by the South Tyrolean Extension Service for fruit and wine growing) were chosen. Furthermore, a representative selection of different altitude levels (200–900 m.a.s.l) and the vicinity to different habitats (apple orchard, fellow, woodland, vineyard, water canal) were considered. The cultivars were restricted to the main *Malus* × *domestica* varieties grown in the surveyed region, namely Golden Delicious and Gala. In 2014, a total of 44 orchards were monitored and in subsequent years (2015, 2016), the number of orchards increased to 50 orchards, most of which were managed according to the guideline of integrated production. In the intensively managed orchards, weed control programs, such as mulching, tillage, as well as the application of herbicides, were used to manage the orchard floor vegetation.

### 2.2. Collection of Fulgoromorpha and Cicadomorpha

To assess the presence of Auchenorrhyncha species, a semi-quantitative approach was applied using (I) the beat tray sampling technique on the canopy layer (CL), and (II) the sweep netting technique (round sweep-net: open diameter: 33 cm) in order to investigate the fauna present in the understory vegetation under the apple trees [43,44,45]. The beat traying was carried out in a seven to 14 days rhythm from end of February until the end of October and by sampling 100–200 randomly chosen branches (one branch per tree) per site and day. Sweep netting was carried out four times a year (150 strokes/orchard) by walking in a straight line between the tree rows of the apple orchards (TR), covering approximately 75 m of the riparian vegetation of the orchard floor per site at each sampling point. The vegetation coverage of TR was quite heterogeneous for each site and at each sampling point (40–90%). In 2015 and 2016, the sweep-net sampling was extended to the ecotone (EC) of apple orchards, i.e., the vegetation between the cultivated zone and adjacence habitats, such as hedgerows, ditches, or meadows. It was carried out four times a year for each site (150 strokes/sampling point). Sampled insects were transported to the laboratory and stored at −80 °C. Morphological identification was performed according to the mainly male-based keys provided by Mazzoni et al. 2001 [46], della Giustina 1989 [47], Ossiannillson [48,49,50], Holzinger et al. 2003 [51], and Biedermann and Niedringhaus 2004 [52].

### 2.3. Faunistic Analysis

Based on the species composition data, index value of diversity, evenness, and dominance were calculated for each zone (CL, TR, and EC) and year.

The dominance index (D_i_) represents the percentage contribution of each species on total catches [53,54] calculated as

D_i_ = (n_i_ × 100)/N, (1)

where n_i_ = Number of individuals of the species i, N = Total individuals of all species.

The categorization of dominance classes followed Engelman (1978) [53]. The frequency (F) provided information about the distribution of one species in the sampled area and was calculated according to Mühlenberg et al. 1993 [54]:

F = (G_i_ × 100)/S, (2)

where G_i_ = number of site records for a species i; S = number of all sites surveyed.

Furthermore, to describe the biodiversity the diversity index Shannon–Wiener index (HS) [54]

HS = −∑(p_i_ × log p_i_); p_i_ = n_i_/N, (3)

and the evenness (E)

E = HS/HS_max_; HS_max_ = −log(1/I), (4)

where I = number of species detected.

For each zone and year, in order to characterize and compare the species, richness and equitability [54,55] were calculated.

Due to the high number of captured species, the subsequent molecular PCR analysis were restricted to “dominant” and “subrecedent”/ “recedent” species (i.e., species with a D_i_ of ≥2 in DL or a D_i_ ≥3 registered in TR and EC) and species that exceeded a calculated frequency (F) of 15%. Furthermore, species that have been previously mentioned as possible or confirmed vectors of phytoplasmas were analyzed as well.

### 2.4. Extraction of DNA from Different Insect Species

Based on the defined criteria (see above) selected species were subjected to subsequent qPCR analysis. DNA from single individuals was extracted using the DNeasy^®^ 96 Blood & Tissue Kit (Qiagen, Hilden, Germany). Unless otherwise noted, all buffers, racks, and tubes mentioned below are part of this extraction kit. The extraction procedure was a combination of the QIAGEN Supplementary Protocol DY14 (“Purification of total DNA from insects using the DNeasy^®^ Blood & Tissue Kit”; August 2006) adapted to the DNeasy^®^ 96 Blood & Tissue Kit as follows: For DNA extraction, 180 µL phosphate buffered saline (PBS, pH 7.2; 50 mM potassium phosphate, 150 mM NaCl; not part of the DNeasy^®^ Blood & Tissue Kit) and a 3-mm diameter tungsten bead (Qiagen, Hilden, Germany; not part of the DNeasy^®^ Blood & Tissue Kit) were added to every collection microtube containing an insect specimen. The samples were disrupted for 3 min at 30 Hz in the Tissue Lyser II (Qiagen, Hilden, Germany), and the lysates were collected by a short spin centrifugation of the 96-well microcentrifuge plate. Then 20 µL Proteinase K and 200 µL buffer AL (without ethanol) were added, vigorously mixed, and incubated for 10 min at 56 °C, and 200 µL 96% (v/v) ethanol (not part of the DNeasy^®^ Blood & Tissue Kit) was added to each sample and processed following the instruction of the manufacturer.

### 2.5. Detection and Quantification of ’Ca. P. mali’

DNA integrity of each sample was checked by amplifying the 28S rDNA D9–D10 divergent domain (fragment number V) using two primers published by Dietrich et al., 2001 [56]. The PCR mix contained 1× Green GoTaq^®^ reaction buffer (Promega, Milano, Italy), 800 µM dNTP-mix (i.e., a mix 200 µM of each dNTP), 0.7 µM forward, and 0.7 µM reverse primer, respectively, and 0.01 U/µL GoTaq^®^ polymerase (Promega, Milano, Italy). The final reaction volume was 10 µL, containing 2 µL undiluted DNA sample. The cycling conditions were 3 min initial denaturation at 95 °C and 35 cycles of denaturation for 1 min at 95 °C, 1 min annealing at 60 °C, and 1 min elongation at 72 °C, followed by a final elongation step at 72 °C for 5 min. Each PCR product was loaded on a 1% agarose gel stained with ethidium bromide, and samples showing an amplicon at about 760 bp were considered suitable for a reliable phytoplasma diagnostic.

The detection and quantification of ’ *Ca*. P. mali’ in the insect samples was either performed with a SYBR based quantitative PCR using specific primers for the phytoplasma specific rpl22 gene as described in Monti et al. 2013 [57] or with a quantitative PCR approach using phytoplasma specific 16S rDNA specific primers and a TaqMan™ hydrolysis probe (Thermo Fisher Scientific, Monza, Italy) described by Baric and Dalla Via 2004 [58]. Both PCR approaches showed similar detection levels for ’ *Ca*. P. mali’ (data not shown). The reason why either SYBR or the hydrolysis probe approach was performed for a certain insect species was based on the spike results as described below in this paragraph. The SYBR PCR was performed as follows: 1× KAPA SYBR^®^ FAST qPCR Master Mix (Kapa Biosystems; Hoffmann-La Roche, Basel, Switzerland) was mixed with 0.25 µM primer rpAP15f-mod and 0.25 µM primer rpAP15r3 [57] in a total volume of 10 µL containing 2 µL undiluted template DNA. The samples were amplified using the following thermal cycling program: 20 s initial denaturation at 95 °C, 35 cycles of 3 s denaturation at 95 °C, and 30 s primer annealing at 60 °C. The amplification was followed by amplicon denaturation from 65–95 °C (0.5 °C/cycle; 5 s) for melting curve analysis. The setup for the quantitative PCR using specific primers and the hydrolysis probe described in Baric and Dalla Via 2004 [58] contained 1 X iQ™ Multiplex Powermix (Bio-Rad, Hercules, CA, USA) 0.9 µM primer qAP-16S-F, 0.9 µM primer qAP-16S-R, and 0,2 µM of the probe qAP-16S (labeled with FAM). The total PCR mix volume was 10 µL containing 2 µL undiluted DNA template. The cycler program comprised 3 min initial denaturation followed by 35 cycles of denaturation at 95 °C for 15 s and 1 s annealing at 60 °C. The samples of a PCR run were only evaluated when the PCR-efficiency was 100% ± 5% and R2 ≥ 0.99. These values were obtained from the standard curve of each individual run. Every sample, standard, and control were always measured in technical triplicates. Positive samples showed a melting curve peak similar to the positive control. The phytoplasma titer was quantified based on the four-point plasmid standard curve analyzed in parallel with the samples in each PCR run. Due to size and morphological differences between the tested insect species, the DNA yield and the potential presence of PCR inhibitors might vary strongly in DNA extraction samples from different insect species [59]. To verify that the diagnostic detection of ’ *Ca*. P. mali’ in each insect species was reliable, a set of at least seven individuals (tested negative regarding phytoplasma presence) per species was analyzed using spike-controls. These control samples were spiked with a defined number of a plasmid carrying the phytoplasma specific PCR target. The subsequent quantitative PCR analysis of these spiked control samples should reveal the amount of the previously spiked plasmid. Each species was initially spike-tested using SYBR-based quantitative PCR. However, SYBR analysis is error-prone if high concentrations of double-stranded DNA are present in the sample background [59]. In case no reliable phytoplasma detection was achieved in the SYBR spike-controls, the analysis was repeated using the 16S specific primers, the hydrolysis probe, and the respective plasmid, as described above. Based on the spike results, either SYBR or the hydrolysis probe quantitative PCR analysis was applied to the respective insect species batch. AP phytoplasma concentration in each specimen was expressed as phytoplasma/insect. Based on the linear detection range (determined with the plasmid standard curve) of the qPCR run, a sample was defined as positive and quantifiable if ≥75 copies/PCR were detected. In a PCR sample, 2 µL genomic DNA were used from a total of 100 µL/insect. The limit to define an insect positive can thus be translated into ≥3750 phytoplasma/insect. Results were compared to phytoplasma quantities in *C. picta* (n = 348) and *C. melanoneura* (n = 205) sampled in the same area [60].

## 3. Results

### 3.1. Auchenorrhyncha Species Present in South Tyrolean Apple Orchards

**Figure 1:**
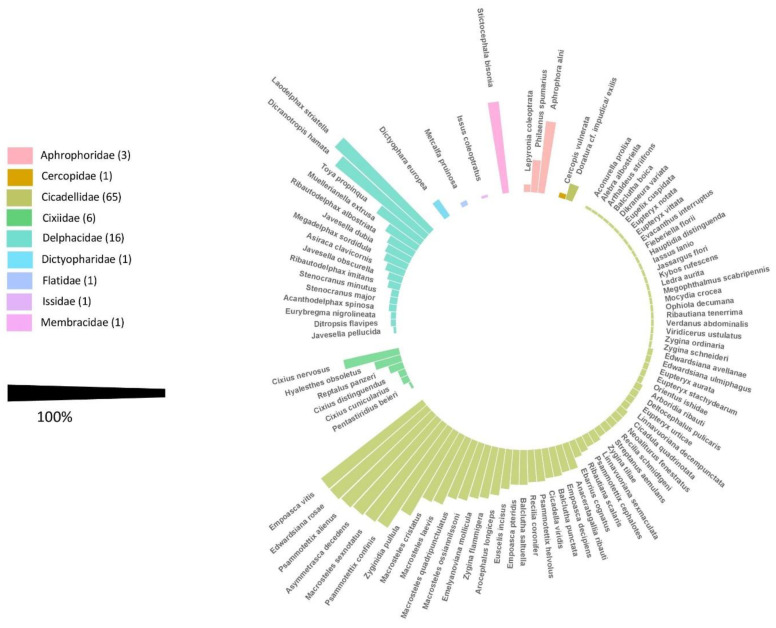
Overall frequency (F mean) of each Auchenorrhyncha species recorded during the whole study period in the agroecosystem “apple orchard”.

During the three-year survey, 31,485 adult Auchenorrhyncha were collected. In total, 23,552 specimens were morphologically identified to species level, comprising nine families and 95 species (Figure 1; for detailed information see Table S2). The Fulgoromorpha were represented by the family Cixiidae (six species), Delphacidae (16), Dictyopharidae (1), Flatidae (1), and Issidae (1); and the Cicadomorpha by Aphrophoridae (3), Cercopidae (1), Cicadellidae (65), and Membracidae (1).

In general, seven species, i.e., *Laodelphax striatella* (Fallén) (26.45% of total catches), *Empoasca vitis* (Göthe) (23.05%), *Edwardsiana rosae* (Linnaeus) (9.39%), *Psammotettix alienus* (Dahlbom) (7.85%), and *Macrosteles sexnotatus* (Fallén) (7.80%) accounted for more than 60% of total captures in the agroecosystem “apple orchard”. Eight species were detected in more than 90% of investigated sites: *Asymmetrasca decedens* (Paoli), *Dicranotropis hamata* (Boheman), *Ed. rosae*, *E. vitis*, *M. sexnotatus*, *P. alienus*, and *Psammotettix confinis* (Dahlbom).

Fourteen new species could be detected that have so far not been documented on South Tyrolean territory, namely *Stenocranus major* (Kirschbaum), *Arboridia ribauti* (Ossiannilsson), *A. decedens* (Paoli), *Balclutha boica* Wagner, *E. ulmiphagus* Wilson and Claridge, *Fieberiella florii* (Stål), *Kybos rufescens* Melichar, *Megophthalmus scabripennis* Edwards, *Orientus ishidae* (Matsumara), *Viridicerus ustulatus* (Mulsant and Rey), *Zygina flammigera* (Geoffroy) *Zygina ordinaria* (Ribaut), *Zygina schneideri* (Günthart), and *Zygina tiliae* (Fallén) (Table S2).

By sampling the canopy layer (CL) of apple orchards, the presence of 31 species has been identified, largely represented by sporadically captured species (D_i_ ≤ 0.32%, Table 1 and Table S3). Only a few can be categorized as main species (D_i_ ≥ 3.2), and these accounted for more than 90% of total catches in CL: *Aphrophora alni* (Fallen) (D_i_ mean = 5.19%), *A. decedens* (D_i_ mean = 7.03%), *Ed. rosae* (D_i_ mean = 22.49%), and *E. vitis* (D_i_ mean = 59.28%). Most recorded species exhibit mesophilic requirements [61].

The number of recorded species in the understory between the tree rows (TR) captured by sweep netting varied between 37 in 2014 and 51 in 2016, whereas the proportion of sporadically occurring species (D_i_ < 0.3, according to Engelmann 1978 [53]) increase from 2015 to 2016. *L. striatella* (D_i_ mean = 42%), *D. hamata* (D_i_ mean = 10.6%), *M. sexnotatus* (D_i_ mean = 13.5%), *P. alienus* (D_i_ mean = 8.21%), *P. confinis* (D_i_ mean = 5.8%), and *Zyginidia pullula* (Boheman) (D_i_ mean = 4.71%) can be considered as the main species in the understory of the orchards and are described as phloem-feeding insects [61] (Table 1 and Table S3).

In 2015 and 2016, the investigation of the “Auchenorrhyncha” community was extended to the ecotone (ET) of apple orchards, i.e., the transition zone between the cultivated and non-cultivated area, where 67 species were identified across the study period. More than 30% of species records occurred sporadically in the ecotones of investigated apple orchards. *Dicranotropis hamata* (D_i_ mean = 7.23%), *L. striatella* (D_i_ mean = 41.54%), *M. sexnotatus* (F mean = 76%; D_i mean_= 10.06%), *P. alienus* (F mean = 83%; D_i_ mean = 19.79%), and *P. confinis* (F mean = 75%; D_i_ mean = 8.08%) can be considered as main species of the investigated ecotones (Table 1 and Table S3).

The Shannon–Wiener Index (HS) [54], for the monotop “apple” (canopy layer: CL) varied from 1.07 (2014) to 1.36 (2015). The calculated HS mean for TR amounts to 2.02 (SD = 0.45) and was comparable to the evaluated HS mean for ET (HS mean = 1.95, SD = 0.32). The calculated evenness (E) was between 0.36 for CL and 0.54 for TR (Table 1 and Table S4).

A database analysis revealed that 29 of the identified species have been already mentioned as competent or potential vectors of phytoplasma- induced diseases [33].

### 3.2. Detection of ’Ca. P. mali’ in Selected Auchenorrhyncha Species

**Figure 2:**
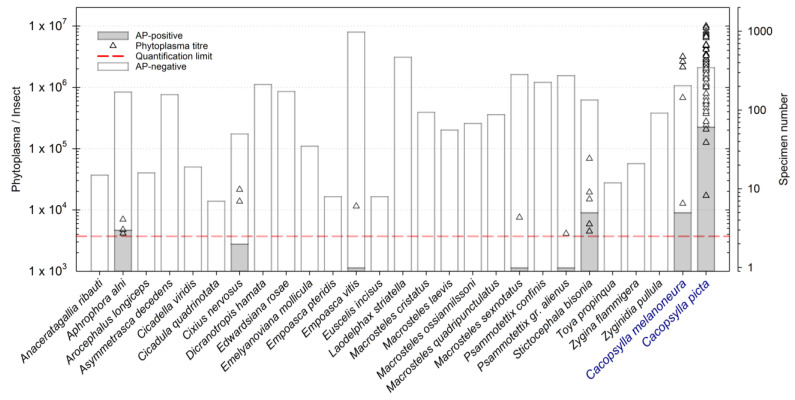
Results of the phytoplasma quantification in selected Auchenorrhyncha species. Quantification was performed by qPCR. The number of tested individuals per species ( *x*-axis) is indicated by the bar plot (right *y*-axis). The grey colored part of the bars indicates the number of AP-positive individuals. The triangles are referring to the left *y*-axis and provide information about the phytoplasma concentration in the AP-positive individuals of the respective species. The red dashed line indicates the phytoplasma detection limit.

Twenty-five out of 95 species met the internally defined criteria regarding frequency and dominance-structure (described in the material and methods section) or have been described to have a potential role in phytoplasma transmission (Table S2). These species were chosen for further laboratory analysis. The qPCR was performed with 3672 individuals (16% of total catches), and ’ *Ca*. Phytoplasma mali’ was detected in 13 individuals belonging to six different species (Figure 2): *S. bisonia* (five out of 135 specimens positive; max = 68,503 phytoplasma/insect), *A. alni* (three out of 170 specimens; max = 6965 phytoplasma/insect), *Cixius nervosus* (Linnaeus) (two out of 50 individuals; max = 21,452 phytoplasma/insect), *E. vitis* (one out of 980 specimens; 11,466 phytoplasma/insect), *M. sexnotatus* (one out of 284 specimens; 7525 phytoplasma/insect), and *P. alienus* (one out of 275 specimens; 4097 phytoplasma/insect). Only six individuals showed relatively high phytoplasma concentrations between 10,000 and 100,000 phytoplasmas per individual: Three *S. bisonia*, two *C. nervosus*, and one out of 980 tested *E. vitis*.

## 4. Discussion

In South Tyrol (Northern Italy), the population densities of *Cacopsylla picta*, the main vector of ’ *Ca*. P. mali’, decreased in the last years, and in some years low densities did not correlate with the high disease incidence observed in apple orchards. This observation led to the assumption that other, yet unidentified insect vectors play an additional role in pathogen transmission [30]. Up until now, insect vectors of phytoplasma disease are found exclusively in the suborder Auchenorrhyncha (Insecta: Hemiptera) [31,32,33]. Thus, the question arose if further insect vectors in this suborder are possibly involved in spreading AP-phytoplasma in South Tyrol. To address this principal question, the following aims were defined for this study: (i) determination of Auchenorrhyncha species present in South Tyrolean apple orchards and definition of a subset of these species that might potentially be involved in AP transmission, and (ii) the establishment of a high-throughput PCR method to analyze and quantify ’ *Ca.* P. mali’ in different insect species in order to analyze a high, representative number of individuals of these insects regarding the presence of AP phytoplasma.

The first step involved an area wide survey regarding the species composition of Cicadomorpha and Fulgoromorpha species and a faunistical analysis of the agroecosystem “apple orchard”. From the canopy layer (CL), the floor vegetation between the apple tree rows (TR), as well as from the nearby ecotones (EC), 95 species have been recorded. Orchard systems are perennial habitats, and the entomofauna richness is higher than in annual crops [62]. Furthermore, they are multi-strata habitats, composed of arboreal strata and the grassy ground, as well as the nearby surroundings. Contributing factors such as land use intensity, pesticide application, exposition, and elevation affect the habitat and, indirectly, influence the species richness [45]. Furthermore, the presence of weeds, especially plants belonging to the family of Poaceae, affect both Auchenorrhyncha species richness and population density, as demonstrated by Thanou et al. [63] for citrus orchards. Thus, a large portion of detected species in the agroecosystem occurred sporadically (<0.32%). Obviously, the Shannon–Wiener index (HS), an approach to describe the biodiversity [54], was lower for the monotop “apple” (CL, HS max= 1.2) than for the ecotones (ET, HS max = 2.1). The calculated evenness (E) for the individual zones was in the medium range, suggesting an unevenly distribution of collected individuals among the registered species-number: The main species (D_i_ > 3.2%) accounted for more than 80% of collected individuals for each investigated zone, indicating the surveyed orchard system as an extreme and biased habitat [53,54]. Consequently, most species, showing high abundances in this agroecosystem are pioneer or synanthropic species, commonly found in disturbed sites [61]. Nevertheless, 14 species so far not detected in South Tyrol could be identified (Table S2).

Several potential, as well as some confirmed, insect vectors of different phytoplasmas that cause diseases that are relevant for South Tyrolean agriculture could be identified: *Asymmetrasca decedens* (vector of ’ *Ca*. Phytoplasma prunorum’) [64], *Anaceratagallia ribauti* (Ossiannilsson) (vector of ’ *Ca*. Phytoplasma solani’) [65], *Euscelis incisus* (Kirschbaum) (vector of ’ *Ca*. Phytoplasma solani’) [66], or *Reptalus panzeri* (P. Löw) (vector of ’ *Ca*. Phytoplasma solani’) had been found [67]. Moreover, the presence of the mosaic leafhopper *Orientus ishidae* (Matsumura) was documented, an invasive species from Asia that has been described as a vector of Flavescence dorée-phytoplasma [68,69] (for detailed information see Table S2).

When selecting species for qPCR analysis (to detect possible ’ *Ca*. P. mali’-infected individuals), the faunistic analysis of the apple orchard was taken into account, without considering the feeding habit of the respective insect but rather their abundances in the surveyed sites. From 3672 analyzed specimens comprising 25 different Auchenorrhyncha species, the presence of AP phytoplasma was detected in only 13 individuals from six different species. The detection of pathogen DNA in an insect is an important first indication if this insect plays a role during AP phytoplasma transmission, but it does not proof its vectoring ability [70]. Several barriers during feeding, multiplication, and movement of the bacterium from the gut and hemolymph to the salivary gland have to be overcome (reviewed in [71]). In addition, the acquisition process period, e.g., the feeding duration, as well as the concentration in the plant itself, may affect phytoplasma acquisition by an insect [3,5]. In a competent vector, phytoplasma acquisition is followed by pathogen multiplication and translocation to the salivary glands to reach a concentration in the insect’s saliva that is sufficient to infect another host plant during feeding [72]. The quantity of the pathogen in individuals of a certain insect species indicates the likelihood of successful transmission by the respective insect species [73,74]. Quantifying AP phytoplasma in a potential transmitting insect species is thus an important initial step to determine if this species might play a role in pathogen transmission. Pedrazzoli et al. [75] showed that the phytoplasma concentration in *C. picta* and *C. melanoneura* reached >10,000 AP phytoplasma per insect within one to two days after acquisition under laboratory conditions. Taking this phytoplasma concentration as an indicative proxy, six out of 13 tested specimens exceeded this value, namely one *E. vitis*, two *C. nevosus*, and three individuals of *S. bisonia*. These species are described as polyphagous and feed on various woody, deciduous host plants [61]. *Empoasca vitis* was the most common Cicadellidae in the investigated area, detected on apple throughout the season. The planthopper *C. nervous* was found in about 50% of investigated sites. Interestingly, positive individuals of *C. nervosus* were captured exclusively in a site with elevated AP inoculum (>50% of AP-symptomatic trees in 2014, data not shown). The AP positive individuals of *S. bisonia* were all captured in different sites, whereby only one site showed a relatively high infection rate. This treehopper is a quite frequent species in South Tyrolean apple orchards (F = 71%), known to harm plants by the oviposition wound in twigs and younger branches where eggs are inserted [76]. Indeed, this species was already mentioned in the context of AP [35].

In our study, the concentration of phytoplasma detected in AP-positive specimens was much lower than in *C. picta* and C *. melanoneura* caught in the same study region (see Figure 2).

Thus, two hypotheses can be formulated:

**Hypothesis** **1.** The insects are transmitting, but ’ *Ca*. P. mali’ infected individuals were caught before the phytoplasma significantly replicated in the insect. Thus, the concentration of the bacterium was found to be low. The duration of the latent period, i.e., the time from initial acquisition by the insect to the ability to transmit the phytoplasma [21] can be very variable: In acquisition trials, in the laboratory conducted by Pedrazzoli et al. [75], the AP phytoplasma multiplicated in *C. picta*, and *C. melanoneura* within a few days, reaching its maximum after four days (>250,000 copies per insect). *Cacopsylla pruni* (Scopoli), vector of European Stone Fruit Yellows (ESFY), showed an effective latency of eight months [77]. In contrast, in *Macrosteles quadrilineatus* (Forbes), vector of Aster Yellows Phytoplasma, laboratory trials showed a ~100-fold increase of the bacteria in the insect, but growth slowed down after six days [74]. Furthermore, the phytoplasma concentration could vary among sex [74,78] and might be influenced by biotic and abiotic factors, such as temperature during latency period [79], genetic composition of the individual itself, and the effect of the bacteria on the fitness of its insect host [80].

**Hypothesis** **2.** The insect is not transmitting and only contains a low concentration of phytoplasma acquired by feeding on an infected plant. The phytoplasma is not able to colonize the insect host and thus does not transmit. This can have at least two reasons: (i) the phytoplasma is eradicated by the insect’s immune system; (ii) the host is lacking important factors that are crucial for colonizing different tissues of the insect. For successful transmission, phytoplasmas have to replicate, traverse the gut and salivary gland cells, and reach the saliva to be transmitted to healthy plants [72,81,82,83]. The phytoplasma must pass several cellular barriers during their migration from the gut to the saliva. It has been hypothesized that this passage is mediated by the interaction of certain phytoplasmal factors with specific structures present on the different insect tissues. The antigenic membrane protein (Amp) of onion yellows (OY) phytoplasma interacts with the insect microfilament complex, and this interaction is important for insect vector specificity [84]. If hypothesis 1 is true, natural populations of transmitting insects that fed on infected plants should contain individuals with high and individuals with low phytoplasma concentrations, i.e., individuals in which the phytoplasma already replicated and individuals that just acquired the pathogen with the phloem sap. In other words, at least a few individuals with a high phytoplasma concentration should be found.

Since ’ *Ca*. P. mali’ was only sporadically detected in the analyzed insects other than *C. picta* and *C. melanoneura* and the concentrations in most cases were very low, the second hypothesis seems more likely. To exclude an occasional ingestion of the bacteria during feeding, the vector ability to acquire, carry, and transmit phytoplasma to healthy plants must be proven by laboratory trials [3,85].

Summarizing, there are no indications that other Auchenorrhyncha species present in the apple orchards contribute to AP phytoplasma transmission. This underlines the prevalent role of *C. picta* and *C. melanoneura* as vectoring insects of this pathogen in South Tyrol. It is thus likely that other factors than unknown vectoring insects are attributable to the—in some years—observed discrepancy between low population densities of *C. picta* and *C. melanoneura* but elevated incidences of AP symptomatic apple trees in the following year. Available monitoring tools for psyllids are error-prone and could underestimate the actual insect populations [30,86,87,88]. Transmission of AP phytoplasma can also occur via root bridges [89,90] or through grafting of infected propagation material [91], and might skew the correlation between insect vector presence and the occurrence of symptomatic trees. Furthermore, latent infected apple trees might become symptomatic several years after infection and can thus not be directly linked to the *C. picta* or *C. melanoneura* population of the precedent year [92]. Competing virulent strains [93] could temporarily mask the disease occurrence, leading to a misinterpretation of the situation in the field.

## 5. Conclusions

The faunistic survey provides an in-depth insight into the Auchenorrhyncha community in apple orchards located in South Tyrol (Northern Italy). The presence of 95 different species has been registered, and 14 species were found that have not been previously described to be present in this territory. The study thus provides a basis for a further detailed analysis on anthropogenic and natural factors influencing these agroecosystem [45,94]. Furthermore, the survey revealed the presence of several insect species associated with different phytoplasma diseases. Some of these phytoplasma diseases are a potential threat for the region’s agriculture [8,33].

In the present study, ’ *Ca*. P. mali’ was surveyed in 3672 individuals comprising 25 different species. Phytoplasma DNA was detected just in a few insects and only in low quantities per individual. It was detected in *S. bisonia*, a species that has been already discussed to play a role during AP phytoplasma transmission [35]. Even though no strong evidence has been found that this species is of importance during pathogen dispersal, its possible vectoring ability should be further elucidated by transmission trials. Taken together, the data indicate that in South Tyrol, no other insect vectors of the taxon Auchenorrhycha exist that play a comparable role during AP phytoplasma transmission like the so far known vectors *C. picta* and *C. melanoneura*.

## Figures and Tables

**Table 1:** Results on the faunistic analysis on the multistrata habitat “apple orchard” for each sampling year and zone from 2014 to 2016. CL = canopy layer; TR = understory vegetation; ET = ecotone; D_i_ = dominance index (%); HS = Shannon-Wiener Index; E = evenness (%).

	2014		2015			2016		
Faunistic Analysis	CL	TR	CL	TR	ET	CL	TR	ET
**Orchards [no.]**	44	44	50	50	50	50	50	50
**Method**	beat tray	insect net	beat tray	insect net	insect net	beat tray	insect net	insect net
**at species level [%]**	97.2	74.46	73.1	79.91	74.07	73.14	70.05	64.33
**Main species [D_i_ ≥ 3.2]**	3	6	4	5	5	3	5	6
**Species [D_i:_ 0.32-3.1]**	6	18	5	9	9	7	10	14
**Species [D_i_ < 0.32]**	16	13	22	26	36	20	36	34
**Σ species**	25	37	31	40	50	30	51	54
**HS**	1.07	2.51	1.36	1.62	1.77	1.3	1.94	2.42
**E**	0.33	0.7	0.39	0.44	0.45	0.38	0.49	0.61

## Data Availability

The data presented in this study are available in supplementary material S1 to S4.

## Supplementary Materials

The following are available online at https://www.mdpi.com/2075-4450/12/1/12/s1: Table S1: Characteristics of the Auchenorrhyncha collection sites. Table S2: Cicadomorpha and Fulgoromorpha species records in the agroecosystem “apple orchard” in South Tyrol. Table S3: Mean dominance (D_i_) and frequency (F) for each species recorded at CL (canopy layer), TR (understory vegetation) and EC (ecotone of apple orchards). Table S4: (A–H): Faunistic analysis of the canopy layer (CL), sampling years 2014–2016, the understory layer (TR), sampling year 2014–2016, and the ecotones (EC), sampling years 2015–2016.
